# Anti-Dengue Virus Antibody Avidity Correlates With Protection Against Symptomatic Dengue Virus Infection

**DOI:** 10.1093/infdis/jiaf171

**Published:** 2025-04-03

**Authors:** Isamu Tsuji, David Dominguez, Jonathan Hernandez, Eloi Kpamegan, José Victor Zambrana, Angel Balmaseda, Hansi Dean, Mayuri Sharma, Eva Harris

**Affiliations:** Vaccines Business Unit, Takeda Pharmaceuticals, Inc, Cambridge, Massachusetts, USA; Vaccines Business Unit, Takeda Pharmaceuticals, Inc, Cambridge, Massachusetts, USA; Vaccines Business Unit, Takeda Pharmaceuticals, Inc, Cambridge, Massachusetts, USA; Vaccines Business Unit, Takeda Pharmaceuticals, Inc, Cambridge, Massachusetts, USA; Sustainable Sciences Institute, Managua, Nicaragua; Department of Epidemiology, School of Public Health, University of Michigan, Ann Arbor, Michigan, USA; Sustainable Sciences Institute, Managua, Nicaragua; Vaccines Business Unit, Takeda Pharmaceuticals, Inc, Cambridge, Massachusetts, USA; Vaccines Business Unit, Takeda Pharmaceuticals, Inc, Cambridge, Massachusetts, USA; Division of Infectious Diseases and Vaccinology, School of Public Health, University of California Berkeley, Berkeley, California, USA

**Keywords:** dengue virus, antibody avidity, protection, cohort study, antibody dissociation, antibody affinity maturation, Nicaragua

## Abstract

Antibody avidity is indicative of antibody affinity maturation following virus infection or vaccination. To determine correlation between preexisting anti-dengue virus (DENV) antibody avidity and secondary DENV exposure outcomes, we assessed anti-DENV antibody avidity, represented as avidity index (antibody response/dissociation rate) in sera of Nicaraguan Pediatric Dengue Cohort Study participants prior to symptomatic or inapparent secondary DENV infections. The avidity index was significantly higher in participants who subsequently developed inapparent versus symptomatic infections. Risk factor analysis suggested that odds of inapparent DENV infection increase as avidity index increases. Antibody avidity index is an important parameter for characterizing protective DENV immune responses.

## Teaser

Anti-dengue virus avidity, represented as avidity index (antibody response/dissociation rate), was significantly higher in Nicaraguan Pediatric Dengue Cohort Study participants who subsequently developed inapparent compared to symptomatic infections. Avidity index may be an important parameter for characterizing dengue immune responses.

Dengue is a mosquito-borne disease caused by infection with 4 antigenically distinct dengue virus (DENV) serotypes (DENV-1, DENV-2, DENV-3, and DENV-4), which account for approximately 390 million DENV infections and up to 96 million symptomatic cases annually [1]. Dengue is a complex disease, and outcomes are influenced by virus serotype, prior immunity, and host factors. Multiple arms of the immune system may contribute to protection against disease severity [2].

Neutralizing antibody (nAb) and other antibody (Ab) effector functions are associated with reduced risk of symptomatic dengue [3, 4]. High-affinity-matured Ab-expressing B cells (selected in germinal centers and differentiated to memory B cells) and Ab-secreting plasma cells are associated with enhanced virus neutralizing potency, breadth, and cytotoxicity. Antibody avidity can be used as a surrogate measurement of the degree of Ab affinity maturation. Increased avidity can impact neutralization potency against DENV [5, 6] so may be a parameter by which protective immune responses against DENV infection can be measured.

Methods to assess the degree of Ab avidity (eg, enzyme-linked immunosorbent assay [ELISA] with chaotropic agents or deep sequencing analysis) can be imprecise or have low throughput, limiting their utility for testing large numbers of samples [7]. We previously described a novel, high-throughput assay to infer the avidity of polyclonal Abs against all 4 DENV serotypes using virus-like particles [7]. Using this assay, we demonstrated that a tetravalent dengue vaccine, TAK-003, elicits anti-DENV Ab with high avidity, an indication of vaccine-driven maturation of the humoral immune response [7]. In addition, we have previously noted high correlations between vaccine-driven avidity and complement-fixing Ab response [8].

While Ab avidity can increase following secondary DENV infections [9], our understanding of how DENV-induced immune responses may impact outcomes of subsequent DENV infections is incomplete. The risk of severe dengue is higher in secondary DENV infection [10], and there remains a need to define immunological parameters following DENV exposure that can predict risk of symptomatic dengue during subsequent infection [11] (see Supplementary Materials for further description).

Here, we evaluated the preexisting anti-DENV Ab avidity as a parameter for outcome of secondary DENV exposure. Utilizing our high-throughput avidity assay [7], we evaluated serum samples collected from a subset of participants in the Nicaraguan Pediatric Dengue Cohort Study (PDCS); the longitudinal design enables detection of inapparent and symptomatic DENV infections. We compared the avidity index of anti-DENV polyclonal Abs in samples from participants who developed symptomatic or inapparent secondary DENV infections to evaluate correlation between the avidity index and clinical outcome in presecondary infection samples adjusting for relevant confounders.

## METHODS

### Dengue Infection Determination

Dengue infections were confirmed by reverse transcription-polymerase chain (RT-PCR) reaction, DENV immunoglobulin M capture ELISA, and DENV inhibition ELISA. See Supplementary Materials for additional details.

### Avidity Assay

The anti-DENV Ab avidity assay assessed immunoglobulin G (IgG) from purified plasma samples collected before secondary DENV infections. The assay was conducted with virus-like particles using the Octet HTX system (Sartorius), as reported previously. Avidity index was calculated as binding response divided by the dissociation rate constant (k_off_). See Supplementary Materials for additional details.

### Statistical Analysis

Avidity assay data were converted to log_10_, and Mann-Whitney tests were applied to compare data before symptomatic or inapparent secondary DENV infection, using GraphPad Prism software (version 8.0.0; GraphPad Software, Inc). Univariate and multivariate risk factor analysis was performed using JMP software (version 16.1.0; JMP Statistical Discovery, LLC) to investigate the correlation between avidity index parameters and the outcome of secondary DENV infections. See the Supplementary Materials for additional details.

## RESULTS

**Figure 1.**
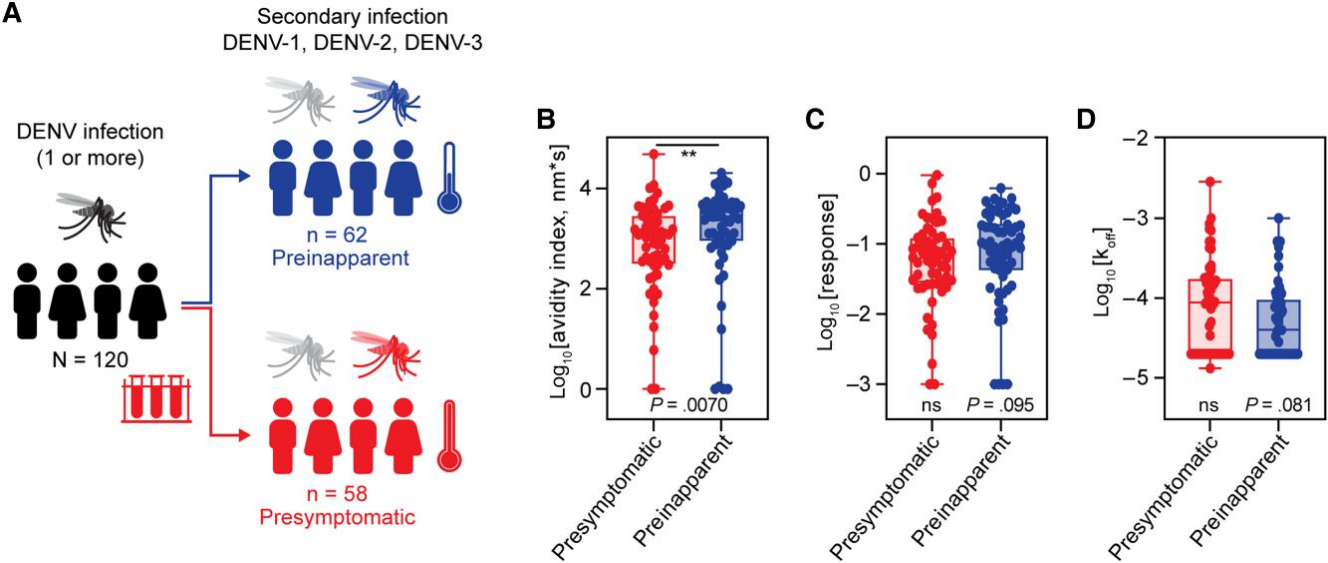
Avidity index of anti-DENV Abs in participants in the PDCS who later developed a symptomatic or inapparent secondary DENV infection. Anti-DENV avidity assays were conducted using purified immunoglobulin G from presymptomatic and preinapparent infection samples. Data were analyzed using the Mann-Whitney test. *A*, Schematic of the participants in the PDCS whose samples are assessed in this study. *B*, Boxplot of the avidity index calculated as binding response divided by k_off_ (nm*s). *C*, Boxplot of the anti-DENV Ab binding response (nm). *D*, Boxplot of the Ab dissociation rate constant, k_off_ (−s). Abbreviations: Ab, antibody; DENV, dengue virus; k_off_, dissociation rate constant; ns, not significant; PDCS, Pediatric Dengue Cohort Study. Box plots: Upper whisker: max value; Lower whisker: minimum value; Box shows 25 to 75 percentiles and median value for centre line.

A subset of 120 PDCS participants with annual samples collected the year before a subsequent symptomatic (n = 58; DENV-1, n = 20; DENV-2, n = 19; DENV-3, n = 19) or inapparent (n = 62; DENV-1, n = 20; DENV-2, n = 22; DENV-3, n = 20) secondary DENV infection from 2005 to 2019 were selected for assessment (Figure 1*A*). Characteristics of participants who later developed a symptomatic or inapparent secondary DENV infection are summarized in Supplementary Tables 1 and 2; all symptomatic infections were dengue fever, according to the 1997 World Health Organization guidelines [11].

The anti-DENV Ab avidity index from prior DENV exposure due to any DENV serotype was significantly higher for participants who later developed an inapparent secondary DENV infection compared to those who developed a symptomatic secondary DENV infection (Figure 1*B*). A representative Biosensorgram shows a higher anti-DENV Ab binding response, a lower k_off_, and a higher avidity index in a participant (No. 114) who later developed an inapparent secondary infection compared with a participant (No. 55) who developed a symptomatic secondary infection (Supplementary Figure 1). This pattern was consistent when data from all participants were included (Figure 1*C* and 1*D*, and Supplementary Table 3).

The availability of infecting serotype information allowed us to specifically assess the anti–DENV-3 Ab response in a subset of participants who later experienced a symptomatic or inapparent secondary DENV-3 infection (n = 19 and n = 20, respectively). Similarly to the combined analysis across all DENV serotypes, the Ab binding response was higher and k_off_ was lower in participants who later developed inapparent infections compared with symptomatic infections (Supplementary Figure 2*A* and 2*B*). Also consistent with the combined analysis, the avidity index was significantly higher in participants who later developed inapparent versus symptomatic DENV-3 infections (Supplementary Figure 2*C*). No significant differences in DENV-3 nAb titers were observed between presymptomatic and preinapparent infection samples (Supplementary Figure 2*D*).

**Figure 2.**
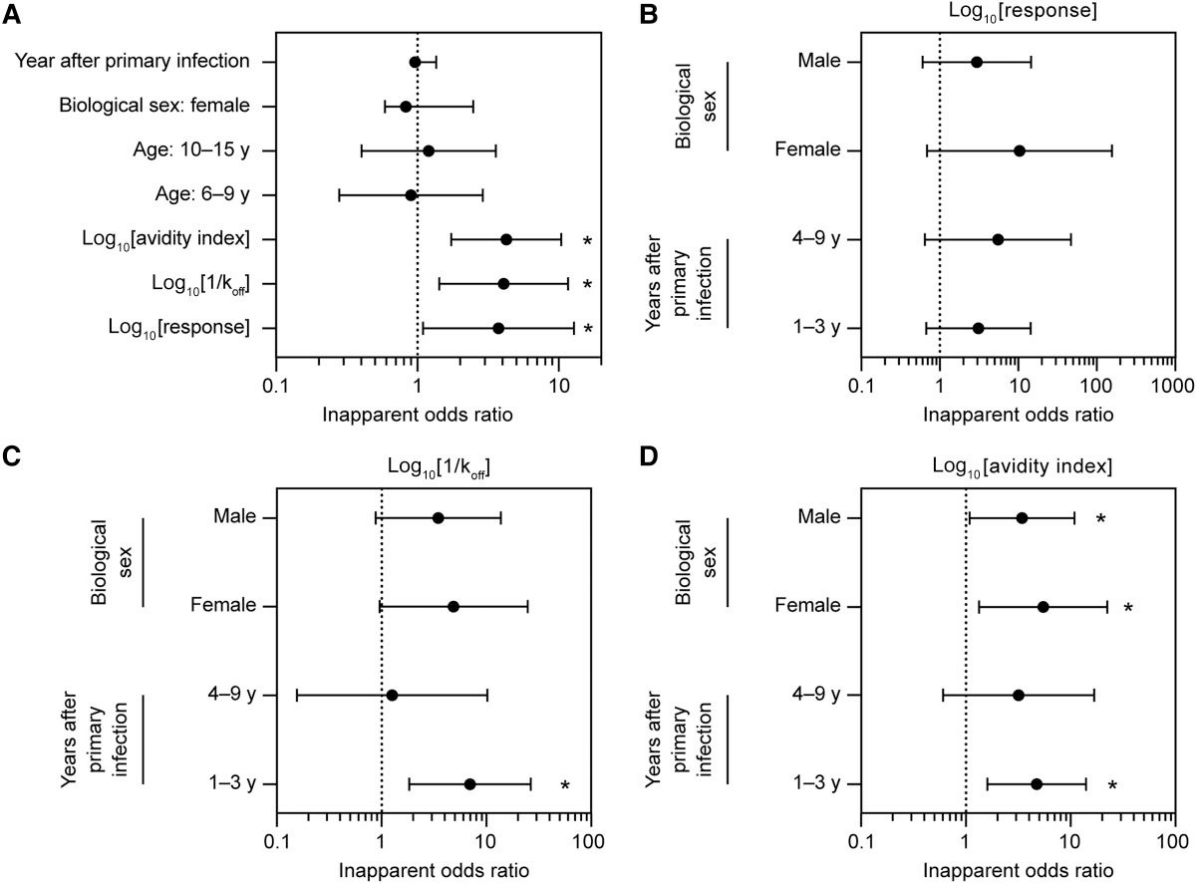
Risk factor analyses. *A*, Factors that may predict the outcome of an inapparent secondary DENV infection. Reference for biological sex, male; reference for age, 3–5 years. *B*–*D*, Risk factor analysis using multivariate regression for the avidity assay parameters of (*B*) binding response, (*C*) k_off_, and (*D*) avidity index. Analyzed factors are years after primary infection (1–3 and 4–9 years) and biological sex (male and female). Participant data with binding response values above half of the lower limit of quantitation were used for the analysis. Odds ratios of log_10_[avidity index], log_10_[1/k_off_], and log_10_[response] are per 1 unit increase. *P < .05. Abbreviations: DENV, dengue virus; k_off_, dissociation rate constant.

Univariate risk factor analysis showed that the preinfection avidity assay parameters of Ab binding response, k_off_, and avidity index were correlated (*P* < .05) with the outcome of secondary DENV infection (Figure 2*A* and Supplementary Table 4). The odds ratios of preinapparent DENV infection (log_10_ scale) for binding response, k_off_, and avidity index were 3.75 (95% confidence interval [CI], 1.10–12.81), 4.07 (95% CI, 1.43–11.61), and 4.25 (95% CI, 1.73–10.42) per 1 unit increase, respectively. The variables of age, biological sex, and years since prior infection did not significantly affect the outcome.

In the adjusted risk factor analysis, biological sex did not significantly affect the outcome for the avidity assay parameters of Ab binding response and k_off_ (Figure 2*B* and 2*C* and Supplementary Table 5). One to 3 years after prior infection, k_off_ and avidity index had a significant impact (*P* = .005 for both) on the outcome of an inapparent secondary DENV infection, with odds ratios of 6.96 (95% CI, 1.82–26.54) and 4.76 (95% CI, 1.61–14.08), respectively (Figure 2*C* and 2*D* and Supplementary Table 5). Interestingly, 4–9 years after prior infection, age had a significant impact (*P* = .015) on the outcome of an inapparent secondary DENV infection (Supplementary Figure 3 and Supplementary Table 5). Overall, we did not observe waning in avidity by years after last infection in a cross-sectional analysis of the preexposure samples analyzed in this study (Supplementary Figure 4).

## DISCUSSION

Although nAb levels are often assessed to evaluate protection against DENV infections, other factors associated with the humoral response, such as Ab maturation, primary versus secondary infection, memory B-cell response, and Ab Fc effector functions, can impact disease outcomes [9, 12, 13]. For instance, we recently reported that Fc biophysical characteristics and effector functions were associated with protection against symptomatic secondary DENV-3 infections and severe dengue disease in PDCS participants [4, 14]. The strength of the Ab response and the antigen-Ab association are important parameters in the polyclonal response to viral infections. Specifically for DENV infection, a study evaluating 108 participants following secondary DENV-3 infections found that serum avidity was associated with the ability to neutralize the virus in certain conditions; significant correlations between IgM avidity and half-maximal neutralizing Ab titer (NT_50_) in acute primary cases, and between IgG avidity and NT_50_ in secondary DENV infections were reported. Additionally, a study in 42 participants assessing Ab avidity following secondary DENV-2 infection demonstrated that a lower Ab avidity at later time points postinfection was associated with increased dengue severity [6].

In our evaluation, for both analyses of all DENV serotype infections and the subset of DENV-3 infections, the avidity index was significantly higher in participants who later developed an inapparent secondary DENV infection compared with those who developed a symptomatic secondary DENV infection. In contrast, this difference was not observed for nAb titers in the subset of participants who developed a secondary symptomatic DENV-3 infection, suggesting that the avidity index was a more sensitive predictor of secondary DENV-3 exposure outcomes. While nAb levels can be associated with DENV outcomes, studies have shown that nAb levels alone may not fully predict the outcome of DENV infections. A recent analysis of the PDCS demonstrated that the correlation between nAb titers and protection against dengue was impacted by other factors such as DENV serotype and previous DENV infections [3]. The reasons for differences in the association of avidity index and nAbs with inapparent versus symptomatic secondary DENV infections are not clear, but we speculate that Ab avidity may be a more sensitive predictor of outcome in DENV response because it reflects the affinity maturation of the Ab response after exposure, which subsequently impacts the quality and breadth of both neutralizing and Fc effector responses to secondary exposure [4, 8]. The divergent trends of nAb magnitude and antibody avidity also highlight the need for assessment of drivers of protective immunity against dengue to expand beyond magnitude and specificity of nAb responses, and to include additional parameters such as Ab avidity and Fc effector functionality in relation to disease outcome.

Risk factor analyses of Ab binding response, k_off_, and the avidity index in determining symptomatic versus inapparent secondary DENV infection indicated that the avidity index was a predictive factor for the outcome of DENV exposure. Of note, multivariate analysis demonstrated that both k_off_ (an indicator of Ab affinity maturation) and avidity index (an indicator of Ab strength) correlated with inapparent DENV infections in the early years (years 1–3) after prior infection but not with inapparent DENV infections in later years (years 4–9). These data suggest that high-affinity mature Ab play an important role in protecting against secondary DENV infections that occur 1–3 years after prior infection (see Supplementary Materials for additional discussion).

Currently, there is no identified correlate of protection for dengue infection and severity, with the challenges being highlighted in a previous review [15]. Few studies have identified a threshold of nAb titers for protection against infection or disease progression for all 4 DENV serotypes [3]. A predictive factor could help identify individuals at risk of developing symptomatic disease and aid in vaccine development. Our results suggest a correlation between the degree of Ab avidity and protection from symptomatic secondary DENV infection due to any DENV serotype. Given recent findings of different protective indicators for each DENV serotype [3], these results demonstrate that additional parameters should be considered for routine humoral response assessments. This and future studies may help place the avidity index as a predictive factor in context with other serological parameters.

## Supplementary Material

jiaf171_Supplementary_Data

## Supplementary Data

Supplementary materials are available at *The Journal of Infectious Diseases* online (http://jid.oxfordjournals.org/). Supplementary materials consist of data provided by the author that are published to benefit the reader. The posted materials are not copyedited. The contents of all supplementary data are the sole responsibility of the authors. Questions or messages regarding errors should be addressed to the author.

## Notes

***Acknowledgments.*** We thank Ankita Sanjali for the data integrity review, Tim Rindfleisch for project management, Andrea Bertolotti-Ciarlet for their suggestions and advice during the studies, and Jaime Cardona-Ospina for his advice regarding statistical analysis. We also thank the researchers of the Vaccine Research and Analytics team at Takeda Vaccine Business Unit, and the participants and investigators involved in the Nicaraguan Pediatric Dengue Cohort Study. Medical writing and editorial support was provided by Excel Scientific Solutions and funded by Takeda.

***Author contributions.*** I. T., E. H., H. D., and M. S. participated in the conceptualization. I. T., D. D., J. H., and J. V. Z. participated in the investigation, methodology, and data analysis. J. V. Z., A. B., E. K., and E. H. participated in the sample selection. I. T., D. D., J. H., and J. V. Z. participated in the data integrity review. I. T., D. D., J. H., and J. V. Z. participated in the visualization. A. B., E. H., H. D., and M. S. participated in the supervision. I. T., D. D., J. H., J. V. Z., A. B., E. H., H. D., and M. S. wrote, reviewed, and edited the manuscript. All authors reviewed the final manuscript.

***Financial support*.** This work was supported by Takeda. The Nicaraguan Pediatric Dengue Cohort Study was supported by the National Institutes of Health National Institute of Allergy and Infectious Diseases (grant numbers P01 AI106695 and U19 AI118610 to E. H., and R01 AI099631 to A. B., and contract number HHSN26620040001C to E. H., Program Director Bruce Birren); the Bill and Melinda Gates Foundation (BMGF) Pediatric Dengue Vaccine Initiative grant VE-1 (to E H.); and the BMGF and Instituto Carlos Slim de la Salud Fighting Infections through Research, Science and Technology grant (to E. H., Program Director Jaime Sepulveda).
