# Supplementary material for: Anti-Dengue Virus Antibody Avidity Correlates With Protection Against Symptomatic Dengue Virus Infection
Source: J Infect Dis. 2025 Apr 3;232(1):e99–e103. doi: 10.1093/infdis/jiaf171 (PMC12308684; doi:10.1093/infdis/jiaf171)
Supplement: jiaf171_Supplementary_Data [file jiaf171_supplementary_data.zip › Supplemental_Material_Avidity_Index_Outcomes_CLEAN 27Feb25.docx]

# Supplementary Materials

## Additional Introduction

The mechanism of severe dengue secondary infection proposes that anti-dengue virus (DENV) antibodies (Ab) in the blood enhance the virus cell entry via cell surface Fcγ receptor, called antibody-dependent enhancement [1]. However, to prevent dengue symptoms, the immune system may contribute to protection against dengue severity [2]. Anti-DENV neutralizing Ab activity to eradicate infected viruses is the most used evaluation of immune response. However, Ab titers do not always correlate with the efficacy outcomes. Increasing evidence suggests that additional aspects of antiviral immune responses may be critical for disease outcomes. Recent studies have focused on the protective effect of Ab-mediated complement and effector-dependent cytotoxicity on flaviviruses [3-6]. Ab affinity maturation leads to higher Ab affinity and avidity, which optimizes antiviral functions, including virus neutralization [7-9], antibody-mediated complements and effector-dependent cytotoxicity [3, 10]. High-affinity matured Abs provide protection from infection and severe symptoms of various viruses, such as DENV, zika and SARS-CoV2 [11-14].

## Materials and Methods

## The Nicaraguan Pediatric Dengue Cohort Study

The Nicaraguan Pediatric Dengue Cohort Study (PDCS) was initiated in 2004 to determine the incidence of dengue virus (DENV) infection and dengue cases, characterize the clinical spectrum of dengue disease, and study viral and immunological determinants of DENV infection outcome. Participants were encouraged to attend the study health center at the first sign of fever or disease symptoms; ~95% of participants complied [15]. Acute blood samples were collected at the first medical consultation (97% within 1‒3 days of illness). Convalescent samples were collected 14‒21 days post-illness onset (94% of cases). Upon medical examination, cases were defined into four categories based on clinical criteria: (A) Individuals meeting the 1997 or 2009 World Health Organization (WHO) case definition for dengue; (B) Individuals with undifferentiated fever of unknown origin; (C) Children presenting with acute febrile illnesses attributed to diseases other than dengue, chikungunya, or Zika; (D) Individuals with other conditions, including rash, that are not associated with fever. A and B cases, and since 2016, rash without fever are tested for DENV diagnosis. Clinical symptoms/signs were recorded using a standardized clinical report form that includes >120 variables [16].

Symptomatic DENV infections were confirmed by (1) detection of DENV RNA using RT-PCR in acute-phase samples [17, 18], (2) virus isolation in acute-phase samples [19], (3) seroconversion in paired acute- and convalescent-phase samples via DENV IgM capture ELISA [20], and/or (4) seroconversion or a >4-fold rise in inhibition ELISA (iELISA) in paired acute- and convalescent-phase samples [16, 21].

Healthy blood samples were systematically collected from all participants annually. Prior to 2016, inapparent DENV infections were defined as seroconversion or a ≥4-fold increase in antibody titer between two consecutive annual samples, as measured by DENV iELISA, in the absence of documented febrile episodes identified as dengue cases within the intervening year [15]. Following the 2016 Zika epidemic, the definition of inapparent DENV infections was revised to include seroconversion or a ≥4-fold increase in DENV iELISA titers in the absence of ZIKV seroconversion, which was assessed via ZIKV nonstructural protein 1 (NS1) blockade-of-binding ELISA or ZIKV iELISA [22, 23]. Participants were considered to have secondary DENV infections if their paired consecutive annual samples had a ≥4-fold increase in iELISA Ab titers, if their serum samples had anti-DENV Ab at enrollment, or if they had a previous documented DENV infection [24].

## Ethics Statement

## The PDCS was approved by the Institutional Review Boards at the Ministry of Health in Nicaragua (protocol: NIC-MINSA/CNDR CIRE-09/03/07-008) and the University of California, Berkeley (protocol: 2010-09-2245). Written informed consent was obtained from a parent or guardian. If the guardian was illiterate, the consent form was read aloud in the presence of a witness, and the guardian’s thumbprint was obtained in lieu of a signature. Assent was obtained from all children aged ≥6 years.

## Antibody Purification

Ab were purified from 50‒100 µL plasma from participants in the PDCS using Protein G Sepharose (Cytiva, Marlborough, MA, USA). After Abs were eluted by glycine-HCl pH 2.7, they were immediately neutralized to pH 7.0‒7.5 by 1M Tris HCl (pH 8.0), and subsequently the buffer solution was exchanged to Dulbecco's phosphate-buffered saline (DPBS, Gibco, Waltham, MA, USA) using Amicon® Ultra-4 centrifugal filter units, MWCO 30 kDa (Merck Millipore, Burlington, MA, USA). Ab concentrations were calculated using the NanoDrop™ 2000 (Thermo Fisher, Waltham, MA, USA): Protein A280 (E1% A280 = 13.8). Ab purity was confirmed by SDS-PAGE (NuPAGE 4‒12% Bis-Tris: Thermo Fisher, Waltham, MA, USA), and Abs with >80% purity were used in the avidity assay.

## Biotinylation of Dengue Virus-like Particles

DENV-1 virus-like particles (VLPs; Nauru/Western Pacific/1974), DENV-2 VLPs (Thailand/16681/84), and DENV-3 VLPs (Sri Lanka D3/H/IMTSSA-SRI/2000/1266) were purchased from The Native Antigen Company (Oxford, UK). These particles expressed pre-membrane (prM) and envelope (E) proteins and displayed detectable prM protein upon sodium dodecyl sulfate-polyacrylamide gel electrophoresis (SDS-PAGE) analysis, indicative of immature state [25]. Biotinylation was conducted by combining the VLPs with a 50 molar excess of EZ-Link™ Sulfo-NHS-Biotin (Thermo Fisher, Waltham, MA, USA) for 60 minutes at room temperature. Excess biotinylation reagents were removed by buffer exchange to DPBS using Amicon® Ultra-4 centrifugal filter units (MWCO 30 kDa). VLPs of the presumed secondary infecting DENV serotype were used to assess the avidity of each participant’s pre-infection serum sample in the avidity assay.

## Avidity Assay

Ab avidity was measured using the Octet HTX systems with Octet Acquisition Software (version 11.1.2.24, Sartorius, Fremont, CA, USA). Biotinylated DENV VLPs representing the presumed secondary infecting serotype (5 µg/mL) in 0.1% bovine serum albumin -phosphate-buffered saline plus 0.05% Tween-20 (BSA-PBST) were captured with a high precision streptavidin (SAX) biosensor (Sartorius, Fremont, CA, USA) for 600 seconds, then 50 µg/mL Biocytin (Thermo Fisher, Waltham, MA, USA) was blocked with excess BSA for 200 seconds. Ab (125 µg/mL) purified from the serum of PDCS participants in 0.1% BSA-PBST was bound to the SAX Biosensor for 1800 seconds. The sensors were then incubated in 0.1% BSA-PBST 0.35 M NaCl for 1200 seconds to dissociate the bound Ab. This assay was conducted at 30°C with agitation (plate shaker, 1000 rpm). The measurement was performed twice, and averaged values were used for further statistical analysis.

Data analysis methods were based on previous reports [26, 27] and optimized for dengue avidity assay. Ab binding response values (nm) for the association phase were recorded for 1800 seconds. The Ab dissociation rate constant (k_off_) from 30 seconds to 600 seconds in the dissociation phase was calculated using the Langmuir 1:1 binding model by Octet Data Analysis Software (version 9.0.0.10; Sartorius). The k_off_ for some serum samples could not be measured due to strong binding; in this case, k_off_ was extrapolated to 2 x 10^-5^ (detectable dissociation from 0–1200 seconds for a 5% signal decrease). Avidity index was calculated as binding response divided by the dissociation rate constant (k_off_). The measurement was performed twice, and average values were used for further statistical analyses. Assay verification and optimization of avidity assay were conducted as previously reported [25]. A review evaluating techniques to assess the binding strength of the Ab responses following vaccination highlights their potential and limitations [28]. Furthermore, the potential limitations of the Octet-based method have been reported previously [29]. However, in this report, we did not evaluate avidity by conventional ELISA but used the Octet-based methods to assess the antibody avidity. k_off_ is the critical factor to reflect antibody maturation [26, 27]. Previously, we reported correlation between k_off_ and Ab binding site amino acid mutations, reflecting the magnitude of affinity maturation of anti-ZIKV monoclonal antibodies [30]. Additionally, we are aware that VLPs may not capture all conformational nuances of a live dengue virus. However, as noted in our previous study [25], DENV VLPs used in the assay showed a dose-dependent association profile with a panel of well-characterized human mAbs and therefore are a conformationally representative and more stable alternative to live viruses for the purposes of the Octet-based assay.

## Statistical Analysis

For avidity assay data analysis, negative to zero response values were extrapolated to 0.001 and avidity index values were extrapolated to 1. k_off_ values for negative to zero response values were extrapolated (1 × 10^–3^ to 5 × 10^–4^) by Octet Data Analysis Software (version 9.0.0.10; Sartorius, Fremont, CA, USA). The analysis included 2 extrapolated datasets for symptomatic secondary infections (1 for DENV-2 infections and 1 for DENV-3 infections), and 3 datasets for inapparent secondary infections (2 for DENV-1 infections and 1 for DENV-3 infections).

Avidity assay data were converted to log_10_, and Mann-Whitney tests were applied to compare anti-DENV Ab avidity parameters (Ab binding response, k_off_, avidity index) and neutralizing Ab titers (for DENV-3 only) from samples collected prior to symptomatic or inapparent secondary DENV infection using GraphPad Prism software (version 8.0.0; GraphPad Software Inc, San Diego, CA, USA). Correlation analysis between Log10 converted avidity index and years after infection was conducted using GraphPad Prism software. The data values greater than half the lower limit of quantitation (LLOQ) were applied and analyzed using a linear regression model. For risk factor analyses, participants who showed Ab binding response values greater than half the LLOQ for DENV-1, DENV-2, and DENV-3 were included; this excluded data from 14 participants who developed symptomatic infections and 14 who developed inapparent infections. k_off_ reciprocal values were used for the analysis, and LLOQ values were estimated as 3.3 times the limit of detection [25]. The analysis was performed using JMP software (version 16.1.0, Cary, NC, USA) to investigate the association between the avidity index and the outcome of secondary DENV infections adjusting for relevant confounders (i.e., age, biological sex, and years after infection). All predictors were also run in separate univariate risk factor analysis.

## Discussion

Multivariate analysis demonstrated that both k_off_ (an indicator of Ab affinity maturation) and avidity index (an indicator of Ab strength) correlated with inapparent DENV infections in the early years (years 1–3) after prior infection but did not correlate with inapparent DENV infections in later years (years 4–9) after prior infection. These data suggest that while high-affinity mature antibodies play an important role in protecting against secondary DENV infections that occur 1–3 years after prior infection. Circulating MBCs at later timepoints may not secrete antibodies with sufficient affinity to protect against secondary infections. Indeed, a preclinical study in mice evaluating how MBCs respond during heterologous second infection reported that high-affinity, long-lived plasma cells and low-affinity MBCs were differentiated for primary flavivirus infections, but during a subsequent heterologous flavivirus challenge, MBCs failed to reenter the germinal center and thereby converted to plasma cells with reduced affinity [31].

## References

1. Changal KH, Raina AH, Raina A, et al. Differentiating secondary from primary dengue using IgG to IgM ratio in early dengue: an observational hospital based clinico-serological study from North India. BMC Infect Dis **2016**; 16:715.

2. Lee MF, Voon GZ, Lim HX, Chua ML, Poh CL. Innate and adaptive immune evasion by dengue virus. Front Cell Infect Microbiol **2022**; 12:1004608.

3. Nascimento EJM, Norwood B, Parker A, Braun R, Kpamegan E, Dean HJ. Development and Characterization of a Multiplex Assay to Quantify Complement-Fixing Antibodies against Dengue Virus. Int J Mol Sci **2021**; 22.

4. Laoprasopwattana K, Libraty DH, Endy TP, et al. Antibody-Dependent Cellular Cytotoxity Mediated by Plasma Obtained before Secondary Dengue Virus Infections: Potential Involvement in Early Control of Viral Replication. The Journal of Infectious Diseases **2007**; 195:1108-16.

5. Agrawal P, Nawadkar R, Ojha H, Kumar J, Sahu A. Complement Evasion Strategies of Viruses: An Overview. Front Microbiol **2017**; 8:1117.

6. Nascimento EJM, Norwood B, Kpamegan E, et al. Antibodies Produced in Response to a Live-Attenuated Dengue Vaccine are Functional in Activating the Complement System. J Infect Dis **2023**; 227: 1282–92.

7. Nakagama Y, Candray K, Kaku N, et al. Antibody Avidity Maturation Following Recovery From Infection or the Booster Vaccination Grants Breadth of SARS-CoV-2 Neutralizing Capacity. J Infect Dis **2023**; 227:780-7.

8. Remmel JL, Frei JC, Butler SE, Lai JR, Ackerman ME. Diverse contributions of avidity to the broad neutralization of Dengue virus by antibodies targeting the E dimer epitope. Virology **2021**; 559:57-64.

9. Tsai WY, Lai CY, Wu YC, et al. High-avidity and potently neutralizing cross-reactive human monoclonal antibodies derived from secondary dengue virus infection. J Virol **2013**; 87:12562-75.

10. Hashimoto G, Wright PF, Karzon DT. Antibody-dependent cell-mediated cytotoxicity against influenza virus-infected cells. J Infect Dis **1983**; 148:785-94.

11. Manuylov V, Burgasova O, Borisova O, et al. Avidity of IgG to SARS-CoV-2 RBD as a Prognostic Factor for the Severity of COVID-19 Reinfection. Viruses **2022**; 14:617.

12. Ravichandran S, Hahn M, Belaunzarán-Zamudio PF, et al. Differential human antibody repertoires following Zika infection and the implications for serodiagnostics and disease outcome. Nat Commun **2019**; 10:1943.

13. Lau L, Green AM, Balmaseda A, Harris E. Antibody avidity following secondary dengue virus type 2 infection across a range of disease severity. J Clin Virol **2015**; 69:63-7.

14. Luo YR, Chakraborty I, Yun C, Wu AHB, Lynch KL. Kinetics of Severe Acute Respiratory Syndrome Coronavirus 2 (SARS-CoV-2) Antibody Avidity Maturation and Association with Disease Severity. Clin Infect Dis **2021**; 73:e3095-e7.

15. Kuan G, Gordon A, Aviles W, et al. The Nicaraguan pediatric dengue cohort study: study design, methods, use of information technology, and extension to other infectious diseases. Am J Epidemiol **2009**; 170:120-9.

16. Katzelnick LC, Gresh L, Halloran ME, et al. Antibody-dependent enhancement of severe dengue disease in humans. Science **2017**; 358:929-32.

17. Lanciotti RS, Calisher CH, Gubler DJ, Chang GJ, Vorndam AV. Rapid detection and typing of dengue viruses from clinical samples by using reverse transcriptase-polymerase chain reaction. J Clin Microbiol **1992**; 30:545-51.

18. Waggoner JJ, Abeynayake J, Sahoo MK, et al. Single-reaction, multiplex, real-time rt-PCR for the detection, quantitation, and serotyping of dengue viruses. PLoS Negl Trop Dis **2013**; 7:e2116.

19. Balmaseda A, Sandoval E, Perez L, Gutierrez CM, Harris E. Application of molecular typing techniques in the 1998 dengue epidemic in Nicaragua. Am J Trop Med Hyg **1999**; 61:893-7.

20. Balmaseda A, Guzman MG, Hammond S, et al. Diagnosis of dengue virus infection by detection of specific immunoglobulin M (IgM) and IgA antibodies in serum and saliva. Clin Diagn Lab Immunol **2003**; 10:317-22.

21. Balmaseda A, Hammond SN, Tellez Y, et al. High seroprevalence of antibodies against dengue virus in a prospective study of schoolchildren in Managua, Nicaragua. Trop Med Int Health **2006**; 11:935-42.

22. Balmaseda A, Stettler K, Medialdea-Carrera R, et al. Antibody-based assay discriminates Zika virus infection from other flaviviruses. Proc Natl Acad Sci U S A **2017**; 114:8384-9.

23. Balmaseda A, Zambrana JV, Collado D, et al. Comparison of Four Serological Methods and Two Reverse Transcription-PCR Assays for Diagnosis and Surveillance of Zika Virus Infection. J Clin Microbiol **2018**; 56.

24. Gordon A, Kuan G, Mercado JC, et al. The Nicaraguan pediatric dengue cohort study: incidence of inapparent and symptomatic dengue virus infections, 2004-2010. PLoS Negl Trop Dis **2013**; 7:e2462.

25. Tsuji I, Dominguez D, Egan MA, Dean HJ. Development of a Novel Assay to Assess the Avidity of Dengue Virus-Specific Antibodies Elicited in Response to a Tetravalent Dengue Vaccine. J Infect Dis **2022**; 225:1533-44.

26. Dennison SM, Reichartz M, Seaton KE, et al. Qualified Biolayer Interferometry Avidity Measurements Distinguish the Heterogeneity of Antibody Interactions with Plasmodium falciparum Circumsporozoite Protein Antigens. J Immunol **2018**; 201:1315-26.

27. Lynch HE, Stewart SM, Kepler TB, Sempowski GD, Alam SM. Surface plasmon resonance measurements of plasma antibody avidity during primary and secondary responses to anthrax protective antigen. J Immunol Methods **2014**; 404:1-12.

28. Klasse PJ. How to assess the binding strength of antibodies elicited by vaccination against HIV and other viruses. Expert Rev Vaccines **2016**; 15:295-311.

29. Kamat V, Rafique A. Designing binding kinetic assay on the bio-layer interferometry (BLI) biosensor to characterize antibody-antigen interactions. Anal Biochem **2017**; 536:16-31.

30. Tsuji I, Vang F, Dominguez D, et al. Somatic Hypermutation and Framework Mutations of Variable Region Contribute to Anti-Zika Virus-Specific Monoclonal Antibody Binding and Function. J Virol **2022**; 96:e0007122.

31. Wong R, Belk JA, Govero J, et al. Affinity-Restricted Memory B Cells Dominate Recall Responses to Heterologous Flaviviruses. Immunity **2020**; 53:1078-94.e7.

**Supplementary Table 1. Characteristics of Participants in the Pediatric Dengue Cohort Study Who Later Developed a Symptomatic Secondary DENV Infection**

|  | Total | Serotype | | |
| --- | --- | --- | --- | --- |
|  | DENV-1, DENV-2, and DENV-3 | DENV-1 | DENV-2 | DENV-3 |
| N (%) | 58 (100) | 20 (34.5) | 19 (32.8) | 19 (32.8) |
| Biological sex, n (%) |  |  |  |  |
| Male | 30 (51.7) | 11 (19.0) | 12 (20.7) | 7 (12.1) |
| Female | 28 (48.3) | 9 (15.5) | 7 (12.1) | 12 (20.7) |
| Age, y, n (%) |  |  |  |  |
| 2‒5 | 8 (13.8) | 1 (1.7) | 3 (5.2) | 4 (6.9) |
| 6‒9 | 20 (34.5) | 5 (8.6) | 9 (15.5) | 6 (10.3) |
| 10‒15 | 30 (51.7) | 14 (24.1) | 7 (12.1) | 9 (15.5) |
| Year of secondary infection, n (%) |  |  |  |  |
| 2005 | 2 (3.4) | 0 (0) | 2 (3.4) | 0 (0) |
| 2006 | 2 (3.4) | 0 (0) | 2 (3.4) | 0 (0) |
| 2007 | 3 (5.2) | 0 (0) | 3 (5.2) | 0 (0) |
| 2008 | 1 (1.7) | 0 (0) | 0 (0) | 1 (1.7) |
| 2009 | 11 (19.0) | 0 (0) | 0 (0) | 11 (19.0) |
| 2010 | 10 (17.2) | 0 (0) | 3 (5.2) | 7 (12.1) |
| 2012 | 19 (32.8) | 19 (32.8) | 0 (0) | 0 (0) |
| 2013 | 2 (3.4) | 1 (1.7) | 1 (1.7) | 0 (0) |
| 2015 | 6 (10.3) | 0 (0) | 6 (10.3) | 0 (0) |
| 2016 | 2 (3.4) | 0 (0) | 2 (3.4) | 0 (0) |
| 2019 | 0 (0) | 0 (0) | 0 (0) | 0 (0) |

Abbreviation: DENV, dengue virus.

**Supplementary Table 2. Characteristics of Participants in Pediatric Dengue Cohort Study Who Later Developed an Inapparent Secondary DENV Infection**

|  | Total | Serotype | | |
| --- | --- | --- | --- | --- |
|  | DENV-1, DENV-2, and DENV-3 | DENV-1^a^ | DENV-2^a^ | DENV-3 |
| N (%) | 62 (100) | 20 (32.3) | 22 (35.5) | 20 (32.3) |
| Biological sex, n (%) |  |  |  |  |
| Male | 35 (56.5) | 8 (12.9) | 14 (22.6) | 13 (21.0) |
| Female | 27 (43.5) | 12 (19.4) | 8 (12.9) | 7 (11.3) |
| Age, y, n (%) |  |  |  |  |
| 2‒5 | 8 (12.9) | 3 (4.8) | 5 (8.1) | 0 (0) |
| 6‒9 | 18 (29.0) | 6 (9.7) | 8 (12.9) | 4 (6.5) |
| 10‒15 | 36 (58.1) | 11 (17.7) | 9 (14.5) | 16 (25.8) |
| Year of secondary infection, n (%) |  |  |  |  |
| 2006 | 8 (12.9) | 4 (6.5) | 4 (6.5) | 0 (0) |
| 2007 | 11 (17.7) | 0 (0) | 11 (17.7) | 0 (0) |
| 2009 | 20 (32.3) | 0 (0) | 0 (0) | 20 (32.3) |
| 2012 | 15 (24.2) | 15 (24.2) | 0 (0) | 0 (0) |
| 2013 | 1 (1.6) | 1 (1.6) | 0 (0) | 0 (0) |
| 2016 | 2 (3.2) | 0 (0) | 2 (3.2) | 0 (0) |
| 2019 | 5 (8.1) | 0 (0) | 5 (8.1) | 0 (0) |

Abbreviation: DENV, dengue virus; iELISA, inhibition enzyme-linked immunosorbent assay; PDCS, Pediatric Dengue Cohort Study.

^a^Infection estimated by iELISA titer and serotype estimated by year of infection.

**Supplementary Table 3. Avidity Assay Data From Pediatric Dengue Cohort Study Participants**

| Participant  No. | Serotype ^a^ | Status | Sex | Years after prior infection | DENV response, nm | DENV k_off_, -s | DENV avidity index, nm*s | DENV-3 NAb titer | ½ LLOQ (response) |
| --- | --- | --- | --- | --- | --- | --- | --- | --- | --- |
| 1 | DENV-1 | Symptomatic | F | 3 | 0.027 | 2.00E-05 | 1360 | NA | ½ LLOQ |
| 2 | DENV-1 | Symptomatic | M | 8 | 0.076 | 2.00E-05 | 3798 | NA |  |
| 3 | DENV-1 | Symptomatic | F | 1 | 0.030 | 2.00E-05 | 1520 | NA |  |
| 4 | DENV-1 | Symptomatic | M | 1 | 0.058 | 1.33E-05 | 4358 | NA |  |
| 5 | DENV-1 | Symptomatic | M | 4 | 0.006 | 2.00E-05 | 300 | NA | ½ LLOQ |
| 6 | DENV-1 | Symptomatic | M | 3 | 0.232 | 2.00E-05 | 11 592 | NA |  |
| 7 | DENV-1 | Symptomatic | M | 1 | 0.007 | 2.00E-05 | 350 | NA | ½ LLOQ |
| 8 | DENV-1 | Symptomatic | M | 4 | 0.048 | 2.00E-05 | 2423 | NA |  |
| 9 | DENV-1 | Symptomatic | F | 2 | 0.024 | 2.00E-05 | 1195 | NA | ½ LLOQ |
| 10 | DENV-1 | Symptomatic | F | 2 | 0.730 | 9.07E-05 | 8057 | NA |  |
| 11 | DENV-1 | Symptomatic | M | 3 | 0.463 | 1.19E-04 | 3903 | NA |  |
| 12 | DENV-1 | Symptomatic | F | 9 | 0.128 | 1.03E-04 | 1241 | NA |  |
| 13 | DENV-1 | Symptomatic | M | 8 | 0.080 | 2.00E-05 | 4008 | NA |  |
| 14 | DENV-1 | Symptomatic | F | 4 | 0.052 | 2.00E-05 | 2578 | NA |  |
| 15 | DENV-1 | Symptomatic | M | 2 | 0.024 | 2.00E-05 | 1210 | NA | ½ LLOQ |
| 16 | DENV-1 | Symptomatic | F | 4 | 0.002 | 2.00E-05 | 98 | NA | ½ LLOQ |
| 17 | DENV-1 | Symptomatic | F | 6 | 0.005 | 8.50E-04 | 6 | NA | ½ LLOQ |
| 18 | DENV-1 | Symptomatic | M | 5 | 0.120 | 2.00E-05 | 6013 | NA |  |
| 19 | DENV-1 | Symptomatic | M | 2 | 0.096 | 4.42E-05 | 2163 | NA |  |
| 20 | DENV-1 | Symptomatic | F | 3 | 0.204 | 2.00E-05 | 10 220 | NA |  |
| 21 | DENV-2 | Symptomatic | M | 4 | 0.104 | 2.39E-04 | 434 | NA |  |
| 22 | DENV-2 | Symptomatic | M | 3 | 0.079 | 1.67E-04 | 472 | NA |  |
| 23 | DENV-2 | Symptomatic | M | 4 | 0.415 | 9.94E-05 | 4176 | NA |  |
| 24 | DENV-2 | Symptomatic | M | 4 | 0.168 | 1.30E-04 | 1290 | NA |  |
| 25 | DENV-2 | Symptomatic | M | 3 | 0.064 | 1.60E-04 | 402 | NA |  |
| 26 | DENV-2 | Symptomatic | F | 7 | 0.268 | 8.52E-05 | 3145 | NA |  |
| 27 | DENV-2 | Symptomatic | M | 1 | 0.038 | 7.05E-04 | 54 | NA |  |
| 28 | DENV-2 | Symptomatic | M | 3 | −0.028 | 1.00E-03 | -28 | NA | ½ LLOQ |
| 29 | DENV-2 | Symptomatic | F | 3 | 0.062 | 1.77E-04 | 352 | NA |  |
| 30 | DENV-2 | Symptomatic | M | 1 | 0.079 | 4.14E-04 | 190 | NA |  |
| 31 | DENV-2 | Symptomatic | M | 1 | 0.962 | 2.00E-05 | 48 090 | NA |  |
| 32 | DENV-2 | Symptomatic | F | 1 | 0.058 | 7.44E-04 | 79 | NA |  |
| 33 | DENV-2 | Symptomatic | F | 1 | 0.055 | 1.50E-04 | 363 | NA |  |
| 34 | DENV-2 | Symptomatic | F | 5 | 0.146 | 1.81E-04 | 808 | NA |  |
| 35 | DENV-2 | Symptomatic | F | 5 | 0.096 | 1.43E-04 | 669 | NA |  |
| 36 | DENV-2 | Symptomatic | M | 4 | 0.236 | 1.56E-04 | 1519 | NA |  |
| 37 | DENV-2 | Symptomatic | M | 1 | 0.027 | 2.00E-05 | 1355 | NA |  |
| 38 | DENV-2 | Symptomatic | F | 2 | 0.137 | 2.21E-04 | 620 | NA |  |
| 39 | DENV-2 | Symptomatic | M | 1 | 0.034 | 4.28E-04 | 79 | NA |  |
| 40 | DENV-3 | Symptomatic | M | 4 | 0.024 | 2.00E-05 | 1178 | NA | ½ LLOQ |
| 41 | DENV-3 | Symptomatic | F | 6 | 0.123 | 3.41E-05 | 3607 | <10 |  |
| 42 | DENV-3 | Symptomatic | M | 5 | 0.036 | 8.69E-05 | 420 | 37 |  |
| 43 | DENV-3 | Symptomatic | F | 2 | 0.026 | 8.95E-05 | 295 | 24 | ½ LLOQ |
| 44 | DENV-3 | Symptomatic | F | 1 | 0.030 | 2.00E-05 | 1515 | 17 |  |
| 45 | DENV-3 | Symptomatic | F | 1 | 0.021 | 2.00E-05 | 1055 | <10 | ½ LLOQ |
| 46 | DENV-3 | Symptomatic | F | 4 | 0.068 | 9.01E-05 | 759 | 36 |  |
| 47 | DENV-3 | Symptomatic | F | 1 | 0.031 | 1.92E-04 | 161 | <10 |  |
| 48 | DENV-3 | Symptomatic | M | 5 | 0.000 | 2.90E-03 | 0 | 34 | ½ LLOQ |
| 49 | DENV-3 | Symptomatic | F | 4 | 0.091 | 2.00E-05 | 4538 | NA |  |
| 50 | DENV-3 | Symptomatic | F | 1 | 0.009 | 5.10E-04 | 17 | <10 | ½ LLOQ |
| 51 | DENV-3 | Symptomatic | M | 2 | 0.085 | 4.97E-05 | 1719 | 29 |  |
| 52 | DENV-3 | Symptomatic | M | 3 | 0.109 | 2.00E-05 | 5425 | 13 |  |
| 53 | DENV-3 | Symptomatic | F | 1 | 0.078 | 8.34E-05 | 930 | 29 |  |
| 54 | DENV-3 | Symptomatic | F | 6 | 0.215 | 7.47E-05 | 2874 | 232 |  |
| 55 | DENV-3 | Symptomatic | M | 2 | 0.038 | 1.28E-04 | 292 | 41 |  |
| 56 | DENV-3 | Symptomatic | M | 2 | 0.015 | 5.10E-04 | 29 | <10 | ½ LLOQ |
| 57 | DENV-3 | Symptomatic | F | 1 | 0.045 | 2.58E-04 | 175 | NA |  |
| 58 | DENV-3 | Symptomatic | F | 1 | 0.275 | 1.51E-04 | 1827 | 42 |  |
| 59 | DENV-1 | Inapparent | F | 3 | 0.149 | 2.00E-05 | 7460 | NA |  |
| 60 | DENV-1 | Inapparent | M | 3 | -0.002 | 5.10E-04 | -4 | NA | ½ LLOQ |
| 61 | DENV-1 | Inapparent | M | 4 | 0.024 | 2.00E-05 | 1185 | NA | ½ LLOQ |
| 62 | DENV-1 | Inapparent | F | 4 | 0.184 | 3.34E-05 | 5504 | NA |  |
| 63 | DENV-1 | Inapparent | F | 3 | 0.107 | 2.00E-05 | 5368 | NA |  |
| 64 | DENV-1 | Inapparent | F | 3 | 0.071 | 9.57E-05 | 744 | NA |  |
| 65 | DENV-1 | Inapparent | M | 2 | 0.090 | 2.00E-05 | 4520 | NA |  |
| 66 | DENV-1 | Inapparent | F | 6 | 0.045 | 2.48E-04 | 182 | NA |  |
| 67 | DENV-1 | Inapparent | F | 1 | 0.148 | 1.16E-04 | 1281 | NA |  |
| 68 | DENV-1 | Inapparent | F | 2 | 0.008 | 2.00E-05 | 420 | NA | ½ LLOQ |
| 69 | DENV-1 | Inapparent | F | 3 | 0.326 | 8.75E-05 | 3724 | NA |  |
| 70 | DENV-1 | Inapparent | M | 7 | 0.052 | 7.92E-05 | 655 | NA |  |
| 71 | DENV-1 | Inapparent | M | 1 | 0.088 | 2.00E-05 | 4410 | NA |  |
| 72 | DENV-1 | Inapparent | F | 1 | 0.272 | 2.00E-05 | 13 600 | NA |  |
| 73 | DENV-1 | Inapparent | M | 6 | 0.405 | 2.00E-05 | 20 257 | NA |  |
| 74 | DENV-1 | Inapparent | F | 5 | 0.020 | 2.79E-05 | 719 | NA | ½ LLOQ |
| 75 | DENV-1 | Inapparent | F | 2 | 0.260 | 2.00E-05 | 12 993 | NA |  |
| 76 | DENV-1 | Inapparent | F | 3 | 0.448 | 3.94E-05 | 11 372 | NA |  |
| 77 | DENV-1 | Inapparent | M | 2 | 0.240 | 2.00E-05 | 12 020 | NA |  |
| 78 | DENV-1 | Inapparent | M | 2 | 0.000 | 3.40E-04 | 1 | NA | ½ LLOQ |
| 79 | DENV-2 | Inapparent | M | 2 | 0.124 | 1.37E-04 | 900 | NA |  |
| 80 | DENV-2 | Inapparent | F | 1 | 0.015 | 2.00E-05 | 755 | NA | ½ LLOQ |
| 81 | DENV-2 | Inapparent | F | 1 | 0.008 | 5.10E-04 | 16 | NA | ½ LLOQ |
| 82 | DENV-2 | Inapparent | M | 2 | 0.054 | 2.00E-05 | 2708 | NA |  |
| 83 | DENV-2 | Inapparent | F | 1 | 0.011 | 2.00E-05 | 530 | NA | ½ LLOQ |
| 84 | DENV-2 | Inapparent | F | 3 | 0.184 | 1.61E-04 | 1143 | NA |  |
| 85 | DENV-2 | Inapparent | M | 1 | 0.035 | 1.13E-04 | 308 | NA |  |
| 86 | DENV-2 | Inapparent | M | 1 | 0.298 | 2.00E-05 | 14 915 | NA |  |
| 87 | DENV-2 | Inapparent | M | 2 | 0.188 | 7.55E-05 | 2495 | NA |  |
| 88 | DENV-2 | Inapparent | F | 2 | 0.044 | 2.00E-05 | 2180 | NA |  |
| 89 | DENV-2 | Inapparent | M | 2 | 0.381 | 1.06E-04 | 3577 | NA |  |
| 90 | DENV-2 | Inapparent | M | 2 | 0.082 | 2.00E-05 | 4115 | NA |  |
| 91 | DENV-2 | Inapparent | F | 2 | 0.079 | 2.00E-05 | 3965 | NA |  |
| 92 | DENV-2 | Inapparent | M | 1 | 0.104 | 2.00E-05 | 5222 | NA |  |
| 93 | DENV-2 | Inapparent | M | 2 | 0.094 | 2.00E-05 | 4705 | NA |  |
| 94 | DENV-2 | Inapparent | M | 7 | 0.056 | 2.00E-05 | 2780 | NA |  |
| 95 | DENV-2 | Inapparent | M | 6 | 0.340 | 1.24E-04 | 2743 | NA |  |
| 96 | DENV-2 | Inapparent | M | 1 | 0.175 | 6.34E-05 | 2761 | NA |  |
| 97 | DENV-2 | Inapparent | M | 4 | 0.624 | 9.60E-05 | 6501 | NA |  |
| 98 | DENV-2 | Inapparent | M | 9 | 0.020 | 4.37E-04 | 45 | NA | ½ LLOQ |
| 99 | DENV-2 | Inapparent | F | 7 | 0.442 | 1.17E-04 | 3796 | NA |  |
| 100 | DENV-2 | Inapparent | F | 8 | 0.103 | 1.14E-04 | 907 | NA |  |
| 101 | DENV-3 | Inapparent | M | 6 | 0.282 | 6.84E-05 | 4120 | 28 |  |
| 102 | DENV-3 | Inapparent | M | 6 | –0.002 | 5.10E-04 | -3 | 27 | ½ LLOQ |
| 103 | DENV-3 | Inapparent | M | 6 | 0.313 | 5.66E-05 | 5532 | 23 |  |
| 104 | DENV-3 | Inapparent | M | 6 | 0.069 | 5.26E-05 | 1319 | 109 |  |
| 105 | DENV-3 | Inapparent | F | 6 | 0.022 | 2.00E-05 | 1118 | <10 | ½ LLOQ |
| 106 | DENV-3 | Inapparent | F | 3 | 0.090 | 2.00E-05 | 4520 | 21 |  |
| 107 | DENV-3 | Inapparent | M | 6 | 0.168 | 5.45E-05 | 3073 | 26 |  |
| 108 | DENV-3 | Inapparent | M | 6 | 0.287 | 6.56E-05 | 4366 | 36 |  |
| 109 | DENV-3 | Inapparent | M | 1 | –0.009 | 1.00E-03 | -9 | 16 | ½ LLOQ |
| 110 | DENV-3 | Inapparent | F | 6 | 0.065 | 3.84E-05 | 1703 | 61 |  |
| 111 | DENV-3 | Inapparent | M | 6 | 0.098 | 7.84E-05 | 1256 | 51 |  |
| 112 | DENV-3 | Inapparent | F | 5 | 0.173 | 7.31E-05 | 2367 | 146 |  |
| 113 | DENV-3 | Inapparent | M | 4 | 0.174 | 5.02E-05 | 3472 | 199 |  |
| 114 | DENV-3 | Inapparent | M | 4 | 0.103 | 2.00E-05 | 5140 | 41 |  |
| 115 | DENV-3 | Inapparent | F | 6 | 0.012 | 7.61E-05 | 151 | <10 | ½ LLOQ |
| 116 | DENV-3 | Inapparent | F | 6 | 0.148 | 4.03E-05 | 3677 | 248 |  |
| 117 | DENV-3 | Inapparent | F | 6 | 0.395 | 3.97E-05 | 9971 | 213 |  |
| 118 | DENV-3 | Inapparent | M | 6 | 0.025 | 2.00E-05 | 1270 | <10 | ½ LLOQ |
| 119 | DENV-3 | Inapparent | M | 3 | 0.043 | 2.00E-05 | 2158 | 23 |  |
| 120 | DENV-3 | Inapparent | M | 2 | 0.058 | 2.00E-05 | 2893 | 24 |  |

Abbreviations: DENV, dengue virus; F, female; k_off_, dissociation rate constant; LLOQ, lower limit of quantitation; M, male; NA, not applicable; NAb, neutralizing antibody.

^a^DENV-1 and DENV-2 inapparent infections were estimated by iELISA titer and the serotype was estimated by year of infection.

**Supplementary Table 4. Univariate Analysis of Risk Factors That May Predict the Outcome of an Inapparent Secondary Dengue Virus Infection**

| Factors | N | Odds ratio | Lower 95% CI | Upper  95% CI | *P* value |
| --- | --- | --- | --- | --- | --- |
| Age, y (reference 3–5 y^a^) |  |  |  |  |  |
| 6–9 | 31 | 0.900 | 0.280 | 2.896 | 0.860 |
| 10–15 | 47 | 1.200 | 0.402 | 3.581 | 0.744 |
| Biological sex (reference male^b^) |  |  |  |  |  |
| Female | 55 | 0.827 | 0.403 | 1.697 | 0.604 |
| Years after prior infection | 120 | 1.143 | 0.961 | 1.359 | 0.131 |
| Avidity assay |  |  |  |  |  |
| Log10[response] | 92 | 3.748 | 1.096 | 12.812 | 0.035 |
| Log10[1/k_off_] | 92 | 4.068 | 1.425 | 11.610 | 0.009 |
| Log10[avidity index] | 92 | 4.251 | 1.734 | 10.422 | 0.002 |

Avidity assay data were used over half lower limit of quantitation of response.

Abbreviation: k_off_, dissociation rate constant.

^a^Fourteen participants.

^b^Sixty-five participants.

**Supplementary Table 5. Multivariate Analysis of Risk Factors That May Predict the Outcome of an Inapparent Secondary Dengue Virus Infection**

| Factors | N | Odds ratio | Lower 95% CI | Upper 95% CI | *P* value |
| --- | --- | --- | --- | --- | --- |
| Log10[response] |  |  |  |  |  |
| Time after primary infection |  |  |  |  |  |
| 1–3 years | 53 | 3.105 | 0.673 | 14.328 | .146 |
| 4–9 years | 39 | 5.509 | 0.644 | 47.116 | .119 |
| Biological sex |  |  |  |  |  |
| Female | 41 | 10.371 | 0.689 | 156.056 | .091 |
| Male | 51 | 2.961 | 0.602 | 14.573 | 0.182 |
| Log10[1/k_off_] |  |  |  |  |  |
| Time after primary infection |  |  |  |  |  |
| 1–3 years | 53 | 6.955 | 1.823 | 26.539 | .005 |
| 4–9 years | 39 | 1.256 | 0.154 | 10.216 | .831 |
| Biological sex |  |  |  |  |  |
| Female | 41 | 4.852 | 0.949 | 24.798 | .058 |
| Male | 51 | 3.470 | 0.875 | 13.763 | .077 |
| Log10[avidity index] |  |  |  |  |  |
| Time after primary infection |  |  |  |  |  |
| 1–3 years | 53 | 4.757 | 1.607 | 14.077 | .005 |
| 4–9 years | 39 | 3.198 | 0.606 | 16.879 | .171 |
| Biological sex |  |  |  |  |  |
| Female | 41 | 5.489 | 1.339 | 22.499 | .018 |
| Male | 51 | 3.445 | 1.086 | 10.925 | .036 |
| Age |  |  |  |  |  |
| Time after primary infection |  |  |  |  |  |
| 1–3 years | 53 | 0.929 | 0.779 | 1.109 | .418 |
| 4–9 years | 39 | 1.732 | 1.114 | 2.693 | .015 |
| Biological sex |  |  |  |  |  |
| Female | 41 | 1.181 | 0.931 | 1.45 | .169 |
| Male | 51 | 0.969 | 0.805 | 1.167 | .742 |

Abbreviation: k_off_, dissociation rate constant.

**Supplementary Figure 1.** Representative Biosensorgram of purified immunoglobulin G from 2 participants in the Pediatric Dengue Cohort Study who later developed a symptomatic or inapparent secondary DENV-3 infection. k_off_, dissociation rate constant.


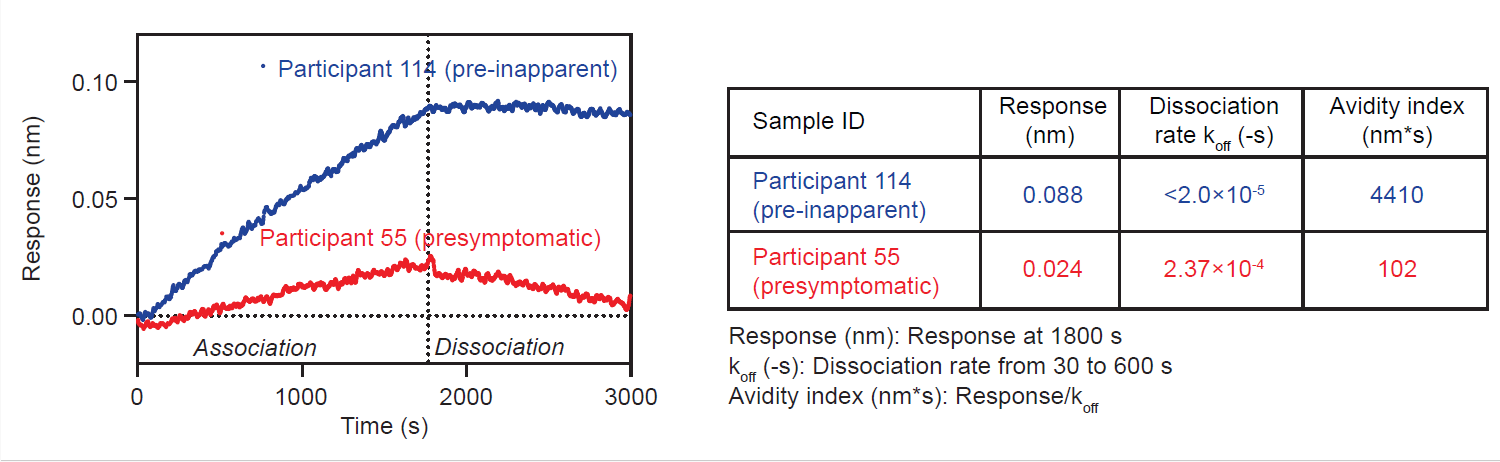


**Supplementary Figure 2**. Anti–DENV-3 antibody response in participants from the Pediatric Dengue Cohort Study who later developed a symptomatic versus inapparent secondary DENV-3 infection. The anti-DENV avidity assay was conducted using purified immunoglobulin G from presymptomatic and pre-inapparent infection samples. Data were analyzed using the Mann-Whitney test. *A*, Boxplot for the anti-DENV antibody binding response (nm). *B*, Boxplot for the antibody dissociation rate constant, k_off_ (-s). *C*, Boxplot for the avidity index calculated as binding response divided by k_off_ (nm*s). *D*, Boxplot for neutralizing antibody titer. DENV, dengue virus; k_off_, dissociation rate constant; N.S., not significant.


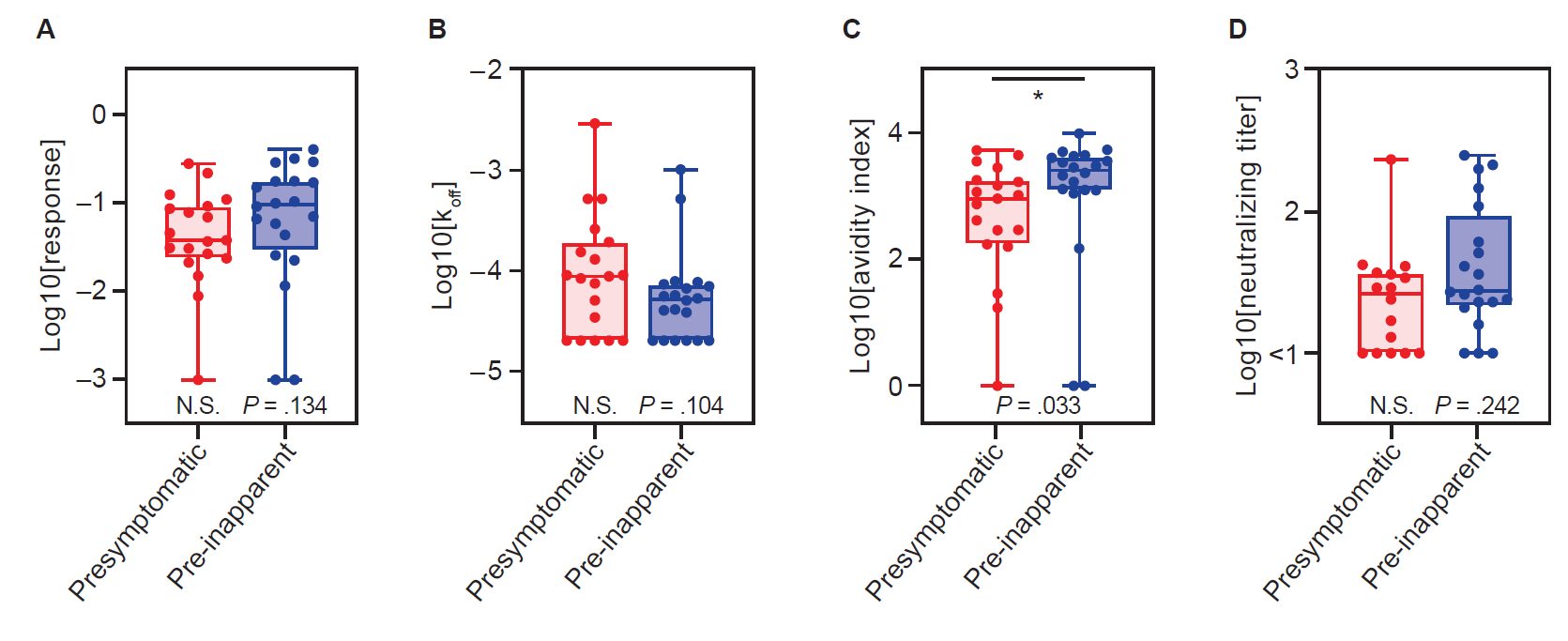


**Supplementary Figure 3.** Risk factor analysis using multivariate regression for age for years after primary infection (1–3 and 4–9 years) and biological sex (male and female). Participant data with binding response values above half of the lower limit of quantitation were used for the analysis.


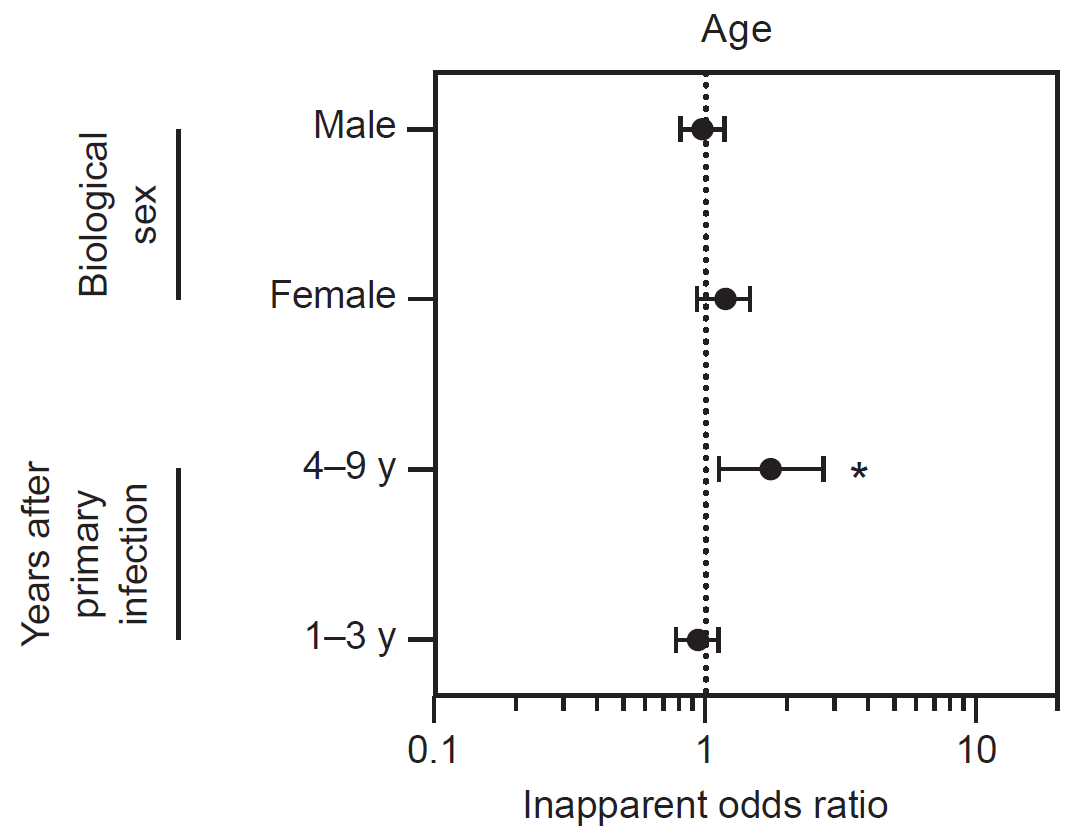


**Supplementary Figure 4.** Correlation analysis between avidity index and years after the primary infections. Log 10 converted avidity index and years after primary infection were plotted. The correlation was analyzed using a linear regression model, and 95% CI values were displayed in the dashed line.
