# Supplementary figures and images for: Anti-Dengue Virus Antibody Avidity Correlates With Protection Against Symptomatic Dengue Virus Infection
Source: J Infect Dis. 2025 Apr 3;232(1):e99–e103. doi: 10.1093/infdis/jiaf171 (PMC12308684; doi:10.1093/infdis/jiaf171)

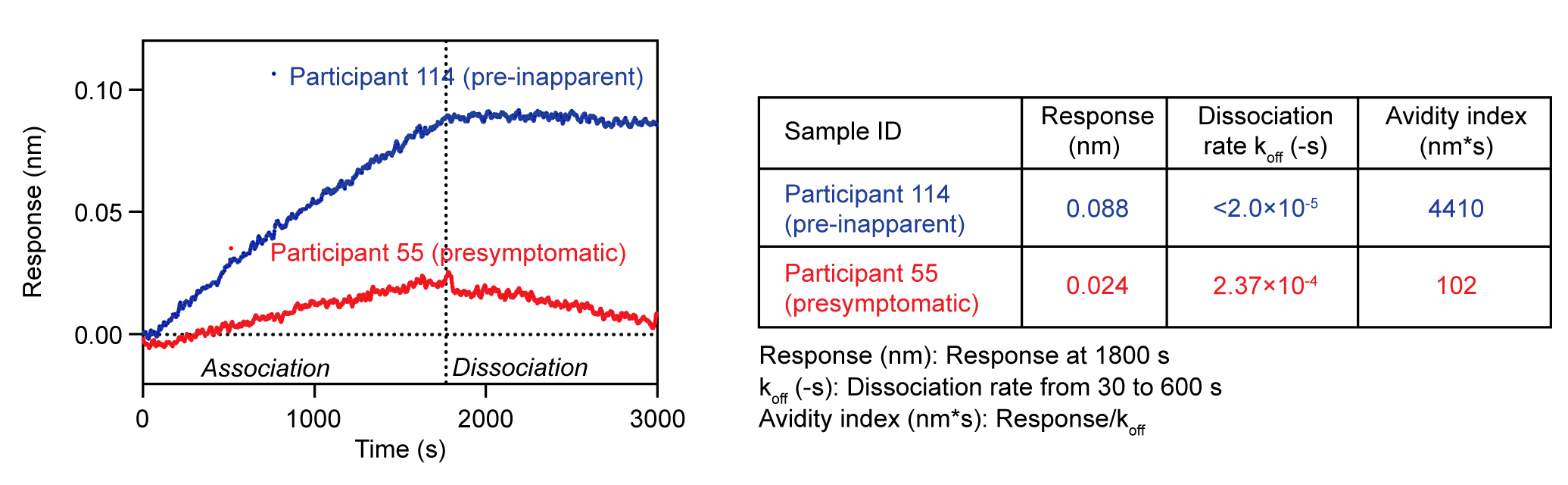

Supplement: jiaf171_Supplementary_Data [file jiaf171_supplementary_data.zip › Avidity SuppFig1.tif]

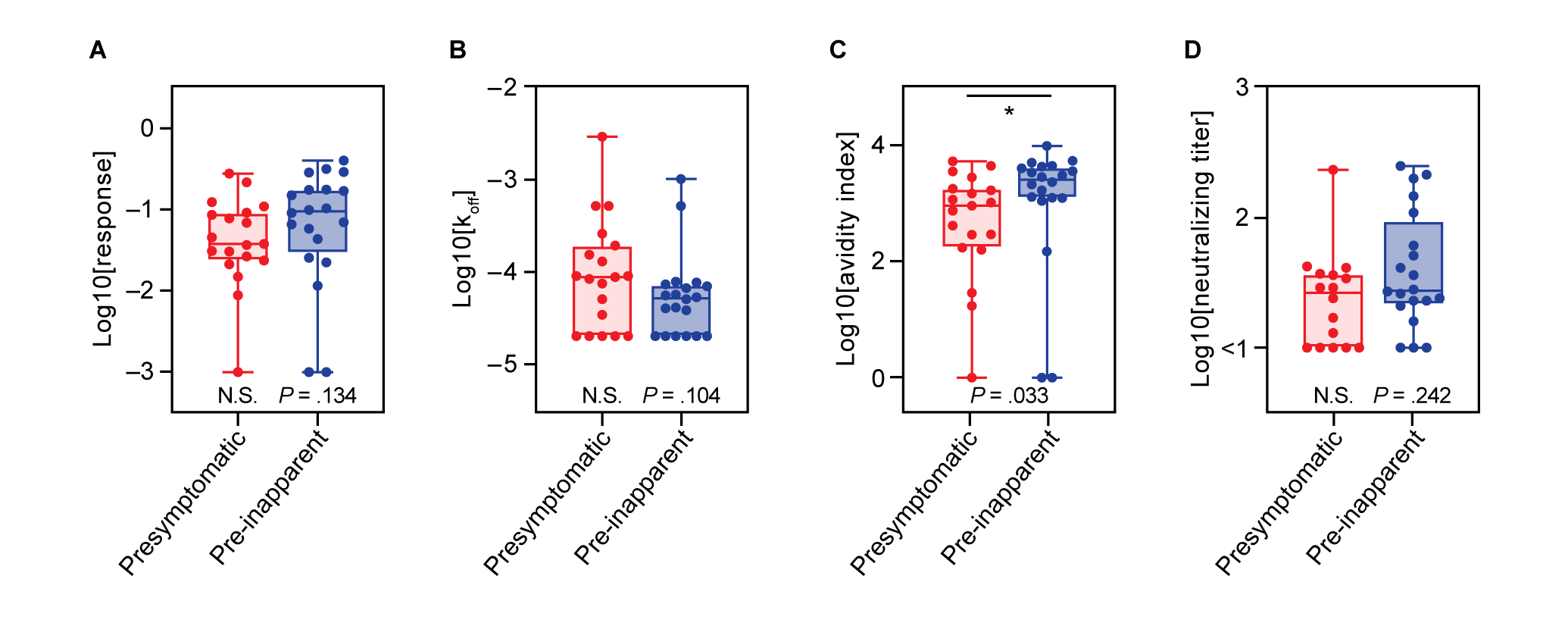

Supplement: jiaf171_Supplementary_Data [file jiaf171_supplementary_data.zip › Avidity SuppFig2.tif]

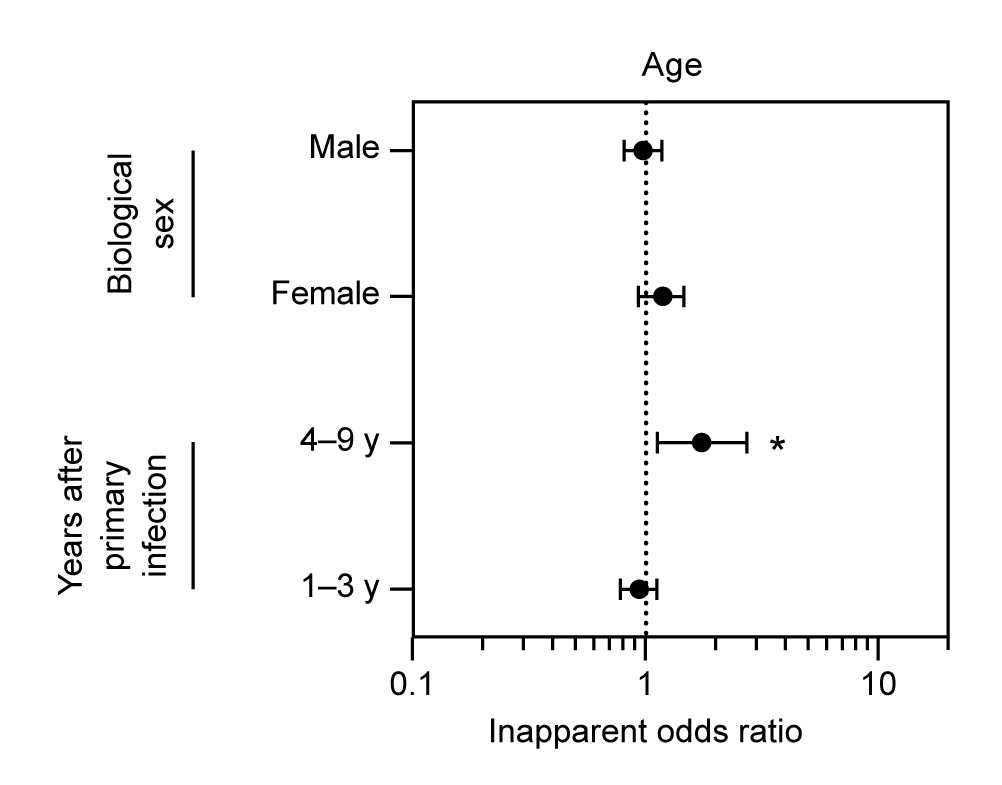

Supplement: jiaf171_Supplementary_Data [file jiaf171_supplementary_data.zip › Avidity SuppFig3.tif]

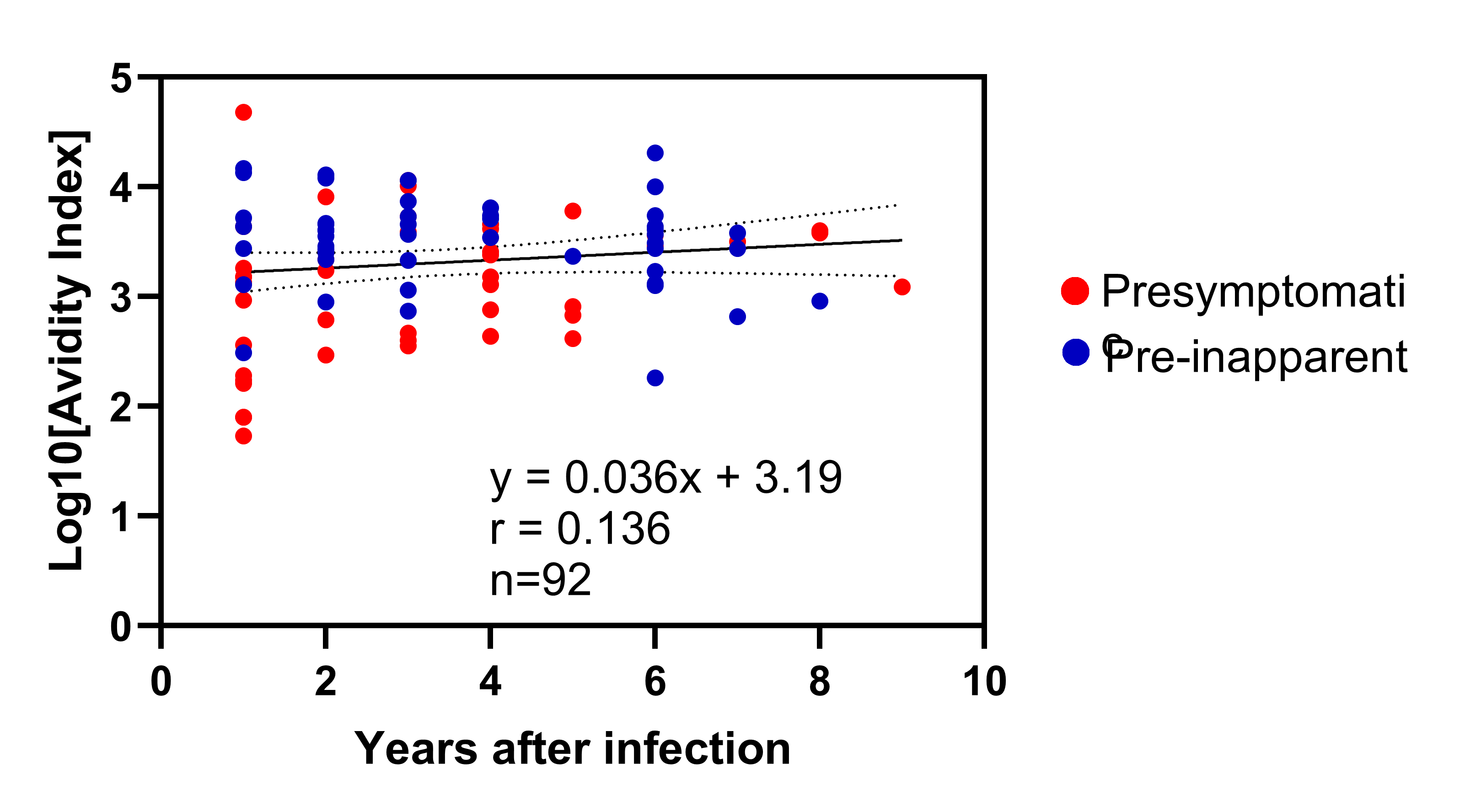

Supplement: jiaf171_Supplementary_Data [file jiaf171_supplementary_data.zip › Suppl Fig 4.tif]
